# The impact of the COVID-19 pandemic on rehabilitation services provided for cochlear implant recipients in Saudi Arabia

**DOI:** 10.1080/07853890.2023.2175902

**Published:** 2023-03-07

**Authors:** Rihab H. Al-Khalil, Batla S. Al-Sowayan, Bayan Albdah

**Affiliations:** a King Abdullah Specialized Children’s Hospital/King Abdulaziz Medical City, Ministry of National Guard Health Affairs, Riyadh, Saudi Arabia; b King Abdullah International Medical Research Center (KAIMRC)/King Saud Bin Abdulaziz University for Health Sciences, Ministry of National Guard Health Affairs, Riyadh, Saudi Arabia

**Keywords:** COVID-19, pandemic, cochlear implant, aural rehabilitation, hearing loss, pediatric, adult

## Abstract

**Objective:**

This study examines the impact of the COVID-19 pandemic on cochlear implantation (CI) recipients in Saudi Arabia. The impact was measured using the results of an online survey that investigated challenges related to access to re/habilitation and programming services, increased dependence on virtual interaction, and emotional impact.

**Methods:**

The cross-sectional online survey reached 353 pediatric and adult CI recipients between April 21st and May 3rd 2020, during the first weeks of implementing the lockdown strategy and the transitioning to virtual settings.

**Results:**

It was revealed that overall access to aural re/habilitation was considerably affected during the pandemic, and that the impact of this disruption was significantly greater for pediatric recipients than for adults. On the other hand, overall access to programming services was not affected. Results also revealed that CI recipients' performance at school or work was negatively impacted by the transition to a virtual communication. In addition, participants noticed a decline in their auditory performance, language skills, and speech understanding. They also registered feelings of anxiety, social isolation, and fear related to sudden changes in their CI function. Finally, the study revealed a gap between CI clinical/non-clinical support provided during the pandemic and the expectations of CI recipients.

**Conclusion:**

Collectively, outcomes from this study highlight the importance of shifting towards a more patient -centered model that offers empowerment and self-advocacy. In addition, the outcomes also emphasize the importance of developing and adapting emergency protocols. This will ensure continuation of services provided to CI recipients during scenario disasters like a pandemic.

**Key messages**
- Pediatric aural re/habilitation was subjected to a significantly greater interruption, compared to adult aural re/habilitation, duringthe COVID-19 shutdown.
- Cochlear Implant (CI) recipients expressed feelings of anxiety, social isolation, and fear. These feelings were related to sudden changes in their CI functioning, caused by interruption of support services during the pandemic.
- Patient -centered model can offer emergency protocols that ensure a smooth continuation of cochlear implant-related re/habilitation and services during disaster scenarios such as the COVID-19 pandemic.

## Introduction

Over 1.5 billion people experience some form of hearing impairment worldwide [1]. For those whose hearing loss cannot be adequately addressed through acoustic amplification alone, cochlear implantat (CI) is a viable option [2]. In Saudi Arabia, the numbers of CI programmes have surged nationwide thanks to their high efficiency and effectiveness [3]. Now, there are about 25 government-supported CI programmes: eight in the Central region, five in the Eastern region, four in the Western region, five in the Southern region, and three in the Northern region. Collectively, these public health sector programmes performed approximately 1000 CI surgical operations in 2018/2019^1^, with an estimated annual growth rate of 15% and success rate of around 97.5% [3,4], which is consistence with CI manufacturers’ reliability reports [5,6]. Alongside CI surgeries, these CI programmes also provide essential services for CI recipients, that are delivered by multi-disciplinary teams including, but not limited to: audiologists, otolaryngologists, speech-language pathologists (SLP), psychologists, social workers, and programme coordinators. In addition, CI devices, consumables, and costs of repair to external items are all covered under the national public health plan. Furthermore, the CI programme teams work closely with CI local distributors to maintain appropriate access to all external items needed by CI recipients.

The majority of CI recipients in Saudi Arabia are pediatrics (over 65%^,^ 18 years and younger), whereas adults (around 30%^,^ over 18 years) constitute a smaller fraction of the total number of people with CI implants^2^. Following successful implantation, CI recipients will requireaural re/habilitation and support services for the rest of their lives, with age-appropriate re/habilitation being a major factor in successful CI outcomes [7]. While the level of re/habilitation and support needed by a CI recipient is dependent on several factors, the most critical and demanding stage for all recipients is the period immediately after implantation. Therefore, during the first two post-implantation years, all CI recipients receive an intensive re/habilitation programme. This involves attending at least two or three aural rehabilitation sessions a week with an SLP. Additionally, the recipient is expected to have regular visits with the audiologist for device programming, these visits are then reduced over time as applicable. Any interruption to the programme will limit the benefits of the CI, particularly for those within the critical age of language acquisition, i.e., ages birth to 5 years.

Immediately after COVID-19 was declared a pandemic on the 11^th^ of March 2020 [8], regulatory bodies in Saudi Arabia took several precautionary measures to contain the spread of the disease. These measures commenced on the 9^th^ and 15^th^ of March, when schools and workplaces closed and were replaced by, where possible, online learning and working from home. For the former, all children and pupils from pre-school age to college level were directed to attend classes through virtual learning hubs. For the latter, workplace attendance was severely restricted in both public and private sectors, with remote-working enforced where possible and with face-to-face meetings replaced with meetings *via* virtual communication platforms. On the 23^rd^ of March a night-time curfew was imposed, followed by travelling restrictions between cities, which in turn was followed by a total lockdown during the Eid holydays in July [9].

Besides the inconvenience of having to adjust to this new isolation lifestyle, the majority of the population quickly adapted to these measures with no significant issues. However, those with special needs, including those with hearing impairments, were faced with considerable obstacles. Certainly, the transition from in-person to virtual communication with others at school, work, or at social events introduced new challenges to the hearing-impaired community. Specifically, people with a CI had to actively adapt to this new method of communication. One major challenge stemmed from the fact that sound transmitters such as headphones used in virtual communication could alter/omit some of the speech signals. Therefore, individuals who use a CI understand less when listening to recorded speech than when hearing live speech [10]. This is all in addition to the loss of visual cues that are crucial for speech understanding, especially when listening without real-time captioning [11]. Individuals with hearing loss, even those with CI, still need visual cues and non-verbal information to fully understand speech, but these are often absent when communicating virtually [12].

Aside from issues surrounding virtual communication platforms, face-to-face meetings during the pandemic also proved very difficult for the hearing impaired and CI recipients – mainly because of face masks. Face masks not only create a visual barrier by obscuring parts of the face that would have provided visual cues, but they are known to alter sound characteristics. Depending on the type of material, face masks can attenuate the sound pressure level (SPL) of the speech signal by up to 29.2 dB, specifically at frequencies of 3000 Hz and higher [13,14]. These visual and acoustic challenges are also exacerbated when social distancing rules require people to stand two metres or more apart. Trecca et al. [15] has investigated this issue and preliminary data revealed that over 85% of individuals with hearing loss faced difficulties with acoustic transmission when facemasks are used during the pandemic.

All these pandemic-related communication barriers can render everyday tasks prohibitively challenging for the hard of hearing [16,17] and can cause profound psychological and emotional stress to CI recipients and their families [18].

In addition to these obstacles, CI recipients also faced disruption to their re/habilitative and programming services, as well as disruption in their communications with their healthcare providers, which often projected to cause adverse clinical side effects [19]. A survey study where children with CI had difficulties attending aural rehabilitations, hardwear services, and intermittency in sound access reported that that this interruption have led to behavioral changes [20]. The same outcome was also reported by Sahoo et al. [21], were parents reported that the pandemic impacted their children’s psychological status and caused behavioral changes.

Therefore, the aim of this study was to investigate the impact of COVID-19 pandemic-related implications on both pediatric and adult CI recipients in Saudi Arabia. The parameters evaluated availability and access to aural rehabilitative and programming services, ease of communication with CI programme staff, and overall provision of support. In addition, this study examines CI recipients’ experience with virtual school/work settings, and how these changes affect their auditory and language performance abilities as well as their mental/emotional wellbeing.

## Study design

### Survey construction

A cross-sectional online survey was created in the Arabic language using Google Doc forms. Prior to distribution, the survey underwent revision and proofreading. It was then completed by three independent test respondents in order to obtain important feedback regarding the level of difficulty and time it takes for the target population (guardians for pediatrics and self-completion for adults) to complete. The survey was divided into 11 sections: the first nine featured multiple-choice questions (in some more than one answer could be selected), and the last two sections contained scale-rating questions. The respondents were able to return to previous sections and change answers prior to submission.

Survey sections covered the following: demographic information (1), CI background information (2), healthcare accessibility and health communication before and after the pandemic (3), aural rehabilitation and programming services prior to the pandemic (4), aural rehabilitation and programming services during the pandemic (5), challenges during the pandemic (6), adaptation of CI recipients during the pandemic (7), clinical and non-clinical organization support during COVID-19 pandemic (8), virtual school/workplace communication (9), CI management and intervention in emergencies (10), digital solutions to improve healthcare services and quality of care (11).

### Distribution and study sample

The survey was distributed between the 21^st^ of April and the 3^rd^ of May 2020, during the first weeks of lockdown and the transition to virtual school and work platforms in Saudi Arabia. Convenience sampling was used to recruit survey participants from all regions of Saudi Arabia. To reach as many targeted individuals as possible, CI associations and support groups were recruited to distribute the link. The Survey link was then sent to participants *via* digital platforms including Twitter and instant messaging applications.

Of the 424 responses to the online survey, 353 (*n* = 326 pediatrics and *n* = 27 adults) were used in this study. Individuals who answered the control question wrong were excluded (71 in total: 51 pediatrics and 20 adults). For the pediatric survey, 197 were completed by fathers, 140 by mothers, and 5 by other family members. All adult participants completed the survey themselves.

### Statistical analysis

Statistical analyses were carried out using (SAS Institute Inc. Cary, 2013) [22]. Descriptive analyses were initially performed to provide information of sample data and visual/practical relevance. To assess the test of independence and correlation, a chi-square correlation test of independence was completed. Where the chi-square test could not be used due to the sample size or distribution concerns, Fisher’s exact test was carried out instead. Statistical significance used in this study was 5%, which was determined as *P* value. Also, missing values in the data were treated as missing completely at random (MCAR). Rating scales were converted from a scale of five to three in all sections for an easier analysis and view of the results.

## Results

### Availability and access to aural rehabilitation, programming, hardware and support services during the COVID-19 pandemic

Two major external factors were considered in the analysis: transportation challenges related to pandemic travel restrictions and economical implications of the lockdown. 50% of pediatric and 30% of adult participants reside in the same city where the speech and language rehabilitation services are located. Moreover, a higher number (around 60%) of pediatric and adult participants reside in the same city where the programming services are located (further socioeconomic and CI background information for all participants is shown in the appendix, Tables 1 and 2). Over 60% of participants reported easy physical access to both rehabilitation and programming services prior to pandemic-related travel restrictions. During the pandemic, travel difficulties and the distance between services and the recipients’ homes were not reported as a major issue.

**Table 1.** Demographic information of pediatric and adult participants; *N* = 353.

Variables		Pediatric % (*n* = 326)	Adults % (*n* = 27)
Age in yrs			
Pediatrics	Adults		
*0–2*	*21–23*	03.9 (13)	14.81 (4)
*>2–4*	*24–45*	18.10 (59)	70.37 (19)
*>4–6*	*45–55*	23.93 (78)	11.11 (3)
*>6–12*	*> 55*	37.12 (121)	03.70 (1)
*13–19*		16.87 (55)	
Gender			
*Male*		51.53 (168)	70.37 (19)
*Female*		48.47 (158)	29.63 (8)
Nationality			
*Saudi*		87.42 (285)	88.89 (24)
*Non-Saudi*		12.58 (41)	11.11 (3)
Marital status			
*Single*			51.85 (14)
*Married*			48.15 (13)
Comorbidities^a,b^			
*Syndromic*		11.35 (37)	18.52 (5)
*Physical*		2.15 (7)	3.70 (1)
*Psychological*		2.45 (8)	7.41 (2)
*Behavioral/mental*		5.21 (17)	0.00 (0)
Medicine^b^			
*Yes*		8.05 (26)	19.23 (5)
*No*		86.38 (279)	80.77 (77)
Residency area			
*Central region*		31.60 (103)	48.15 (13)
*Northern region*		12.72 (40)	7.41 (2)
*Southern region*		14.72 (48)	22.22 (6)
*Western region*		25.15 (82)	3.70 (1)
*Eastern region*		16. 26 (53)	18.52 (5)
Type of residency			
*City*		84.05 (274)	66.67 (18)
*Village/other*		15.95 (52)	33.33 (9)
Housing			
*Owned*		92.02 (300)	40.47 (11)
*Shared*		6.44 (21)	33.33 (9)
*Rented/government or private*		1.53 (5)	25.93 (7)
Educational level			
a. Parents education i. Mother, ii. Father			
*i. No education*		02.15 (7)	
*Elementary/Intermediate school*		10.21 (33)	
*High school*		28.22 (92)	
*Diploma/Bachelor’s degree*		57.36 (187)	
*Master’s/Doctoral degree*		2.15 (7)	
*ii. No education*		1.53 (5)	
*Elementary-Intermediate school*		7.36 (24)	
*High school*		25.77 (84)	
*Diploma/Bachelor’s degree*		58.28 (190)	
*Master’s /Doctoral degree*		7.06 (23)	
b. CI recipients			
*No education*			0.00 (0)
*Elementary/Intermediate school*			11.11 (3)
*High school*			29.56 (8)
*Diploma/Bachelor’s degree*			55.56 (15)
*Master’s/Doctoral degree*			3.70 (1)
Occupation^b^			
a. Parents i. Mother, ii. Father			
*i. Private Sector*		6.77 (22)	
*Government*		15.08 (49)	
*Retired/*		1.85 (6)	
*Unemployed*		76.31 (248)	
*ii. Private Sector*		27.91 (91)	
*Government/South soldier^c^*		61.96 (202)	
*Retired*		7.98 (26)	
*Unemployed*		2.15 (7)	
b. CI recipients			
*Student*			18.52 (5)
*Private Sector*			18.52 (5)
*Government*			33.33 (9)
*Retired*			7.41 (2)
*Unemployed*			22.22 (6)
Monthly household income			
*Less than 5000 SAR*		19.63 (64)	51.85 (14)
*50000–10000 SAR*		33.74 (110)	33.33 (9)
*10000–15000 SAR*		27.30 (89)	11.11 (3)
*More than 10000 SAR*		19.33 (63)	3.70 (1)

^a^More than one option can be selected by participants. ^b^Missing data. CI: Cochlear Implant. ^c^South soldier: A soldier on active duty in the southern borders of Saudi Arabia.

**Table 2.** Cochlear implant history and background information for pediatric and adult recipients; *N* = 353.

Variables		Pediatric % (*n* = 326)	Adult % (*n* = 27)
Age at first implantation in yrs			
Pediatrics	Adults		
*< 3*	*>21–23*	54.6 (178)	14.81 (4)
*>3–5*	*24–45*	29.75 (97)	70.37 (19)
*>5–21*	*> 45*	15.65 (51)	14.82 (4)
Language skills before first CI			
*Prelingual*		91.10 (297)	14.81 (4)
*Post-lingual*		8.90 (29)	85.19 (23)
Number of CIs			
*Unilateral*		57.36 (187)	70.37 (19)
*Bilateral*		42.64 (139)	29.63 (8)
CI duration usage (in yrs)^b^			
*Pediatrics*	*Adults*		
*0–2*	*0-1*	2.77 (9)	7.7 (2)
*>2–4*	*>1–4*	32 (104)	23.08 (6)
*> 4*	*> 4*	65.23 (212)	69.23 (18)
CI usage (hours per day) ^b^			
*< 4 or inconsistent*		15.65 (51)	00.00 (00)
*4–6*		4.29 (14)	13.04 (3)
*6–8*		11.04 (36)	00.00 (00)
*> 8*		69.02 (225)	86.96 (20)^b^
Type of CI manufacturer^b^			
*Advanced Bionics*		6.17 (20)	00.00 (00)
*Cochlear Nucleus*		59.57 (193)	66.67 (18)
*Med-El*		33.02 (107)	33.33 (9)
*Other*		1.23 (4)	00.00 (00)
CI program/center			
*Government hospital*		86.81 (283)	85.19 (23)
*Private hospital*		7.36 (24)	7.41 (2)
*Outside Saudi Arabia*		5.83 (19)	7.41 (2)
AR location^b^			
*Government hospital*		49.39 (161)	51.85 (14)
*Private hospital*		19.94 (65)	07.41 (2)
*CI local distributor office*		2.15 (7)	00.00 (00)
*Comprehensive Rehabilitation center*		2.15 (7)	00.00 (00)
*School*		2.67 (9)	00.00 (00)
*AR at home*		18.10 (59)	00.00 (00)
*None*		5.83 (19)	25.93 (7)
Programming location^a,b^			
*Government hospital*		80.67 (263)	84.62 (22)
*Private hospital*		10.74 (35)	7.69 (2)
*CI local distributer office*		9.20 (30)	11.54 (3)
CI external items/spare parts sources during COVID-19^a^			
*CIP in public sector*		33.44 (109)	29.63 (8)
*CIP but receiving at local distributer office*		6.75 (22)	3.70 (1)
*Donation platforms/CI national associations*		71.47 (233)	3.70 (1)undefined
*Health insurance*		1.84 (6)	3.70 (1)
*Direct purchase*		1.53 (5)	62.96 (17)
*None*		5.83 (19)	11.11 (3)
ALD owned by CI recipients^a^			
*External microphone(i.e., mini mic/Audio link)*		20.25 (66)	25.93 (7)
*Built-in Bluetooth*		16.26 (53)	29.63 (8)
*Roger/FM system*		18.71 (61)	18.52 (5)
*Audio jack/loop system*		9.20 (30)	14.81(4)
*None*		54.91 (179)	29.63 (8)

^a^More than one option can be selected by participants, ^b^Missing data.

CI: Cochlear Implant; CIP: Cochlear Implant Program; AR: Aural Rehabilitation; ALD: Assistive Listening Devices; FM: Frequency Modulation.

Furthermore, around 90% of participants in this study are receiving services for CI surgery and aural rehabilitation (i.e., speech and language therapy and device programming services) in government-run hospitals and government-affiliated centres. The majority of participants are Saudi nationals who are eligible for CI surgery and subsequent rehabilitation care under the free-of-charge national health services. Therefore, any economic implications brought on by the pandemic are not likely to affect how CI recipients access rehabilitation or support services during the pandemic. As a result, questions related to healthcare insurance or changes to the income of participants were not significant to this study.

#### Access to aural rehabilitation services

**Figure 1.**
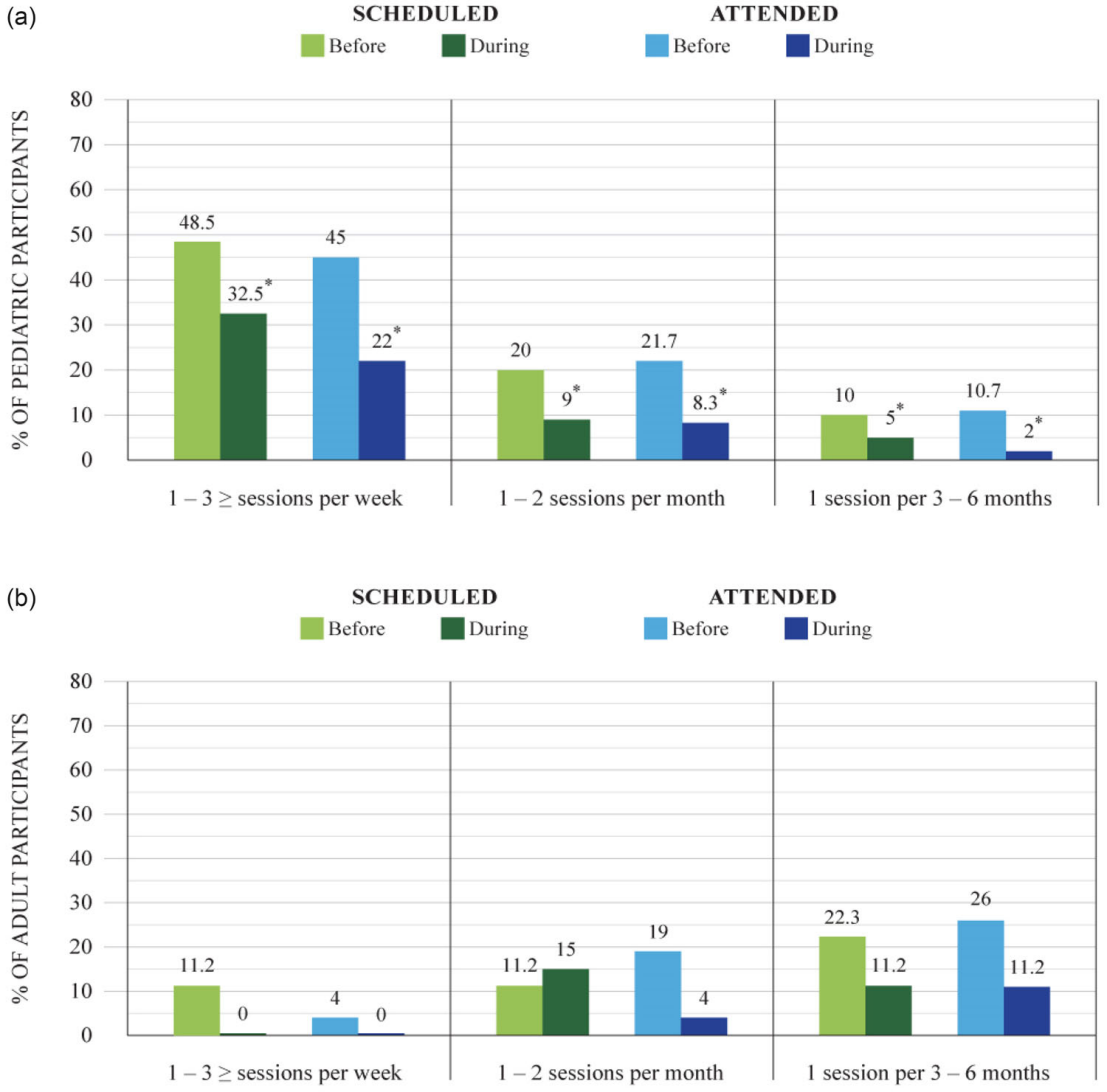
**(a)** Scheduled/attended aural re/habilitation sessions before and during COVID-19 pandemic in paediatrics. *p < 0.001, a correlation test was completed for each subgroup separately for scheduled and attended sessions. Subcategories were categorized as follows: intensive aural re/habilitation (i.e., 1–3 ≥ sessions per week), moderately intensive aural re/habilitation (i.e., 1–2 sessions per month), non-intensive aural re/habilitation (i.e., 1 session per 3–6 months). **(b)** Scheduled/attended aural re/habilitation sessions before and during COVID-19 pandemic in adults. A correlation test was completed for each subgroup separately for scheduled and attended sessions. Subcategories were categorized as follows: intensive aural re/habilitation (i.e., 1–3 ≥ sessions per week), moderately intensive aural re/habilitation (i.e., 1–2 sessions per month), and non-intensive aural re/habilitation (i.e., 1 session per 3–6 months).

Regarding SLP-led aural re/habilitation services, both pediatric and adult participants were asked about the number of scheduled sessions and the number of attended sessions before and during the pandemic (Figures 1(a,b)). The results showed that pediatrics were the most affected in this area (for detailed subgroup analysis, refer to Figure 1(a)). For them, there was a significant drop in the number of scheduled rehabilitation sessions during the pandemic compared with pre-pandemic (Table 3a). There was also an overall decline in the number of scheduled sessions for adults during the pandemic, but statistical analyses did not reveal the decrease to be significant (Table 3a), (for detailed subgroup analysis, refer to Figure 1(b)).

**Table 3.** Correlation test *P* value results for tested parameters for *N* = 353 (Pediatrics: *n* = 326 and Adults: *n* = 27).

a. Correlations for Results 1.1	*p* Value
*Access to aural rehabilitation:*	
*Before pandemic x during pandemic*	
Pediatrics	< 0.0001**
Adults	0.3217
**b. Correlations for Results 1.2**	
*Device programming availability:*	
*Before pandemic x during pandemic*	
Pediatrics	0.0983
Adults	0.298
*Immediate internal CI device emergency services by CIP x region of residency*	
Pediatrics	2.695
Adults	0.4
*Hours of CI use x malfunction of external CI hardware* ^a^	
Pediatrics	0.002*
*Self-rated local distributer accessibility:*	
*Before pandemic x during pandemic*	
Pediatrics	0.9
Adults	0.3705
*Self-rated local distributer support x CI manufacturer type*	
Pediatrics	0.8158
Adults	0.085
**c. Correlations for Results 2**	
*Parental, Self qualifications x use of digital AR platforms*	
Pediatrics	< 0.001**
Adults	0.04*
*Self-rated language skills deterioration x self-initiated home-based aural rehabilitation ^a^*	
Pediatrics	0.049*
*Self-rated school/work support x hearing difficulties in virtual platforms*	
Pediatrics	< 0.001**
Adults	0.0046*
**d. Correlations for Results 3**	
*Self-rated auditory performance deterioration? x time of implantation*	
Pediatrics	0.002*
Adults	0.043*
*Anxiety/fear of sudden change in CI function x time of implantation*	
Pediatrics	0.212
Adults	1
*Feeling socially isolated x self-rated auditory performance deterioration?*	
Pediatrics	0.2910
Adults	0.12
*Feeling socially isolated x health comorbidities ^a^*	
Pediatrics	0.01*

*p <.05. **p<.001. ^a^Correlation test was not administered to adults due to limited participants.

CIP: Cochlear Implant Program; CI: Cochlear Implant; AR: Aural Rehabilitation.

As for the number of attended sessions, in general, participants who were more committed to attending their scheduled sessions pre-pandemic recorded a better attendance record during the pandemic. Again, pediatrics were more affected than the adults as they significantly missed more sessions during the pandemic compared with the number of missed sessions pre-pandemic (for detailed subgroup analysis, refer to Figure 1(a)). Parent survey respondents expressed that the main reason behind missing sessions during the pandemic was the fear of COVID-19 exposure. For adults, statistics comparing pre-pandemic and pandemic attendance patterns showed no significant change, despite an observed overall decrease (for detailed subgroup analysis, refer to Figure 1(b)). However, those adults who did miss their aural re/habilitation sessions also reported the fear of COVID-19 exposure as the main reason. In both groups, the rate of attendance for aural re/habilitation sessions and area of residency did not show a significant correlation.

**Figure 2.**
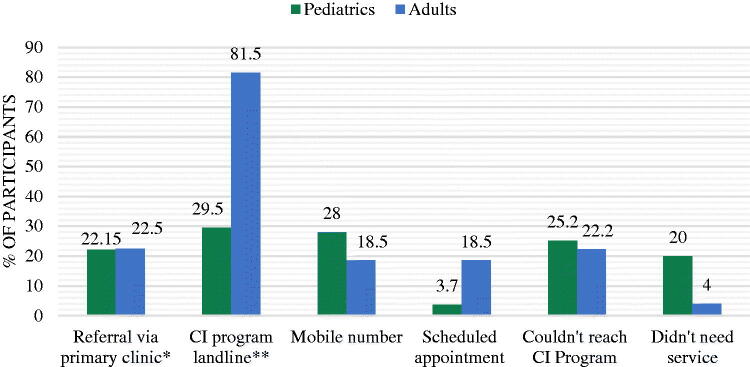
Methods of contacting CI program members in emergencies during COVID-19 pandemic. *Includes scheduled, walk-in, emergency room visits. ** Includes audiologists, and CI program coordinator.

During the period in which the study survey was distributed, a 24-hour lockdown was imposed for the Eid national holidays. During which, Participants attempted to contact the CI Programme *via* different methods (Figure 2). When they were asked if they had emergency access to members of their rehabilitation team during the six days of total lockdown, the survey indicated that pediatrics were overall more affected by restricted communication with their CI team than the adults. Parents of pediatric recipients also reported difficulties reaching out to all members of the team. In terms of which member was reached out to, pediatric survey participants reported that the most reachable CI team members during lockdown were the audiologist and the CI Programme coordinator; the adult group cited audiologists as the easiest to reach.

Finally, participants were asked to rate the overall support received by the CI team during the pandemic. The majority of the pediatric group reported a low satisfaction rate, while most of the adults reported that they were moderately satisfied. When asked which member of the rehabilitation team they felt was most needed during the pandemic, the pediatric group selected their SLP, while the adult group selected their audiologist.

#### Access to programming and hardware services

For on-site programming services, there was no significant change in availability and access before and during the pandemic, according to the pediatric and adult survey results (Table 3b). During the survey period, 7% of CI pediatric recipients received remote programming services through a local CI distributer office visit in their residential area, mostly outside the city where the programming office is located. Participants who received remote programming were able to do so successfully *via* conferencing software at their local CI distributor office. There, a facilitator connected their device to a pod so that the primary audiologist could gain full access to the implant.

For CI internal device emergency services, only 18% in each group required an immediate intervention for issues related to their implanted device. Participants reported swelling, redness, and infection in the implant area as the major issues. Both groups reported that they received adequate urgent care by the CI team regardless of their residential location (Table 3b).

On the other hand, of pediatric and adult participants, 32.21%; 40.74% reported that they experienced malfunctioning or loss of external CI hardware. For pediatric CI recipients, the hours of device usage significantly influenced the rate of malfunction of external items (Table 3b). The external items susceptible to damage, starting with the most prone, were: microphone filters, drying kits, connecting cables, and the rechargeable battery. However, most of the participants reported that they had spare parts at home, while the rest were able to source replacements for the damaged items from elsewhere.

It is worth mentioning that there was no significant correlation in service accessibility at CI local distributer offices before and during the pandemic (Table 3b). When participants were asked to rate the support provided by local CI distributors during the pandemic, the majority of both groups reported a low level of support. Furthermore, a low satisfaction rating was seen across all local distributer offices of all the brands of CI, with no particular brand scoring significantly higher or lower compared with the others (Table 3b).

### Home, school and work performance during the COVID-19 pandemic

**Figure 3.**
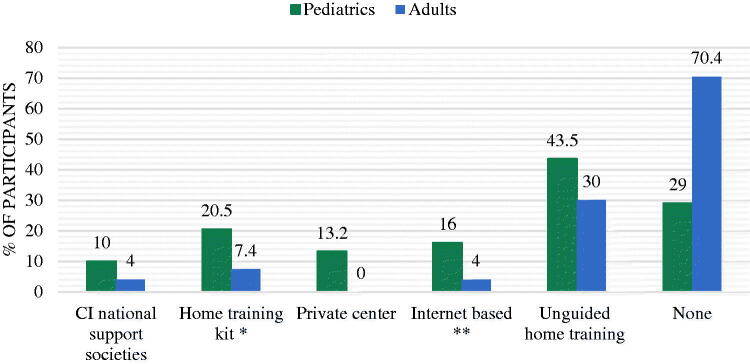
Sources of aural re/habilitation material during COVID-19 pandemic. *By primary SLP. **Includes Google search, social media, aural rehabilitation mobile applications.

**Figure 4.**
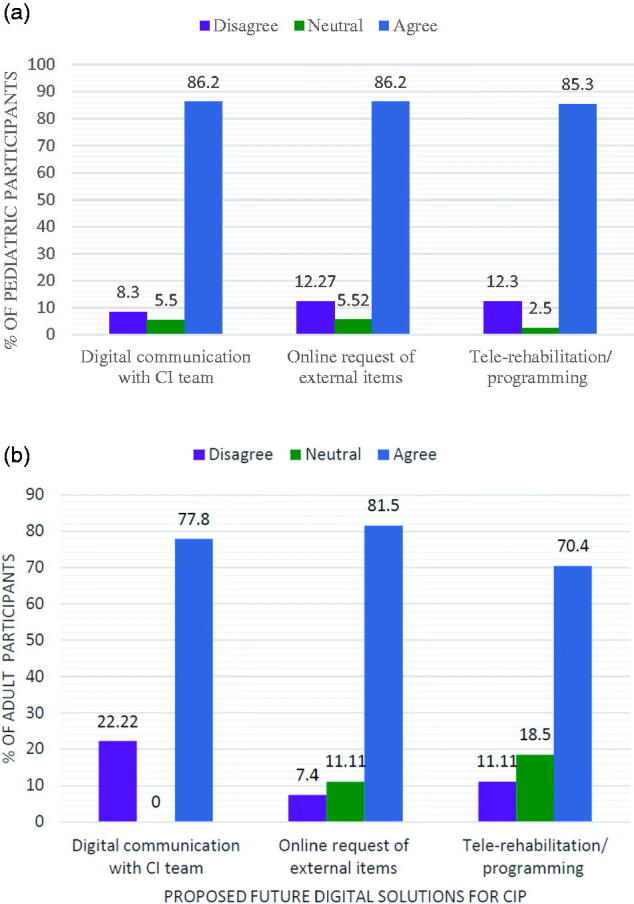
**(a)** Agreement to proposed future digital solutions for CI program in pediatrics. **(b)** Agreement to proposed future digital solutions for CI program in adults.

The survey’s results show that during the pandemic, only 20% of pediatric and 7% of adult CI recipients received a home-based aural re/habilitation training kit from their SLP. Most of the recipients who did not receive the training kit, self-initiated home training by utilizing internet-based platforms and social media (Figure 3). For both groups, there was a significant correlation between parental and self-qualifications and the utilization of digital platforms for training materials (Table 3c). Regarding rehabilitation results, parents reported no noticeable improvement in the language skills of their children with home-based training (Table 3c). Nevertheless, CI participants and their families exhibited readiness to integrate digital solutions into their health care services (Figure 4(a,b)).

Attending school and work prior and during the pandemic was investigated. Fifty three% of participants (Pediatric and adults) attended educational facilities prior to the pandemic. This number dropped to 35% during the pandemic, when virtual attendance was required. Whereas 60% of adult participants were employed and attended work. This number dropped to 48%, when virtual attendance was enforced during the pandemic (refer to Table 1 for details).

Most participants, around 80%, reported hearing difficulties while using a virtual setting, which consequently affected their performance at school/work. Yet, disclosure of hearing difficulties to teacher/employer, utilization of assistive listening devices (ALDs), and implementation of communication strategies while using the virtual platforms were rated very low in both groups. Regarding ALD utilization, even though 45% and 70% of pediatrics and adults, respectively, owned at least one ALD (refer to Table 2 for details), only one third received training on how to use them.

Similarly, participants in both groups rated school/employer support to be either non-existent or low. In addition, results show a significant correlation between reporting low school/work support and self-assessed hearing difficulties (Table 3c). The national CI associations were rated slightly higher in support compared with other sectors.

### Subjective changes in CI recipients’ daily living during the pandemic

Participants were asked to self-assess the challenges they faced. In terms of auditory performance, the majority of participants from both groups reported severe deterioration. There was a significant correlation between the length of time since a participant underwent CI surgery and the magnitude of the deterioration (Table 3d). Those who were implanted just before the pandemic faced more hearing difficulties compared with participants who were implanted earlier and passed the critical stage. As for self-assessed deterioration in language skills, only a few of the pediatric recipients’ parents and almost none of the adults reported any deterioration. Self-assessed deterioration in speech understanding – reported by adults only – was described as either moderate or severe by 45% of participants.

Participants were also asked if they felt that face masks affected their face-to-face communication and understanding of verbal communication. Most parents did not think that face masks had an effect on their children’s communication, while most of the adult participants reported face masks did impact their communication abilities.

When answering questions regarding feeling anxious and socially isolated, one quarter of the pediatrics group and one third of the adults group reported having experienced severe anxiety and fear of sudden changes in the function of their CI during the pandemic. These feelings were not influenced by the length of the time passed since the device was implanted (Table 3d). Extreme feelings of social isolation were also experienced by around 20% of participants in both groups. While data analysis for both groups showed no obvious link between feeling socially isolated and noticing changes in auditory performance (Table 3d), there was a significant correlation in the pediatrics group between feeling socially isolated and having other health comorbidities (Table 3d). Finally, when asked if they believed the emotional impact of the pandemic was greater for a person with hearing loss compared with someone with normal hearing, 30% of pediatrics and over 50% of adults strongly agreed to this statement.

## Discussion

The aim of this study was to examine the impact of the COVID-19 pandemic on CI recipients in Saudi Arabia. More than 350 CI pediatric and adult recipients participated in an online survey. Survey questions addressed aural rehabilitation progression, programming services offered, and availability of hardware maintenance. In addition, participants were surveyed on their experience with changes in communication patterns and support offered to them by their clinical and non-clinical institutions. The study was conducted shortly after COVID-19 was declared as a global pandemic by the World Health Organization.

Participants completed the survey during the two months where the preventive measures were at their highest. Therefore, the impact of the pandemic on participants, including restricted travel and emotional stress, was expected to have been captured at its most severe. Also, the disturbance to the workflow of CI rehabilitation and support teams—was also expected to have been captured at its highest. Physical visits to CI re/habilitation locations (mostly hospitals) were limited to high-risk cases only; for others, care was provided through phone-based medical screenings to address concerns, function, and performance evaluation. In addition, local distributors of CI and other affiliated entities/offices also closed and relied on phone customer service.

Analysis of survey answers revealed that pediatric CI re/habilitation progression was more vulnerable to pandemic-related implications. This has been shown by the drop in scheduled and attended re/habilitation sessions in the pediatric group. Regarding the scheduling of appointments, a possible cause of the sharp drop could be due to prioritization requirements. The majority of CI recipients are prelingual children, of which around 10% are in an intensive stage of an aural rehabilitation programme. For this group, it is crucial to undergo a minimum of two to three sessions per week as they are in a time-sensitive stage of their rehabilitation. Therefore, it was expected that the aural re/habilitation team prioritize CI pediatric recipients undergoing intensive therapy during pandemic restrictions, thus significantly affecting the appointments available for CI pediatric recipients with non-urgent needs. Therefore, limited clinical capacity mandated that only high-risk patients, or those with urgent medical priorities, are seen in person.

Regarding the adults, the overall number of adult recipients is small in comparison. This resulted in no appointment disturbance or shifting of prioritization, and subsequently there were no significant changes to their scheduling pattern. As for appointment attendance, again pediatric were more likely to miss their appointments during the pandemic mainly due to fair of COVID-19 exposure, which is consistent with a previous study from Germany [23]. On the other hand, programming services were not affected, according to the responses given by the participants in both groups. This could be attributed to 90% of participants being implanted for more than a year and had thus reached a stable programming that, in turn, lessened the frequency of required visits [2,24]. It could also be attributed to that fact that a small portion of participants received remote programming services *via* a remote programming system. In remote programming, participants still had to travel to a local CI distributor office to receive the service, but remote programming outside a hospital setting was expected to at least minimize the fear of disease exposure. This reaffirms that, a wider implementation of tele-practice in aural rehabilitation and programming is critical to prevent any future interruption of CI services during challenging circumstances [25,26]. In general, it is projected that the digitalization of health services will prove to be a practical future approach for an efficient and cost-effective healthcare system [27].

As for language skills in pediatrics, home-based aural re/habilitation training kits were only received by a small number of CI recipients. The majority relied on digital platforms to obtain aural re/habilitation training material with no reported improvement in speech and language skills. This could be a result of not knowing where to get/how to perform effective home-training without directions from their SLP. Therefore, not only it is crucial to provide all CI recipients with home-training kits, but it is also vital to create digital training materials that meet the needs of the targeted population – i.e., platforms in the Arabic language – and educate CI recipients on how to choose the ones that best fit their needs.

Participants also reported feelings of anxiety and fear of sudden changes in the function of their CI. Introducing a patient-centred care model in CI programmes – by empowering CI recipients by teaching them to take control of their CI journey – will lead to better performance outcomes [28]. It will also eliminate feelings of fear, anxiety, and frustration that can result from not knowing what to do in case of interrupted contact with the CI team [29]. The decentralization of care and resources offered to patients is thought to improve clinical outcomes, assuming that it will not diminish communication with health care providers [19].

Another important aspect of a patient-centred care model is self-advocacy. Participants reported having hearing difficulties during virtual school/work meetings with no real-time captioning, yet they did not inform their teacher/employer that this was the case. Furthermore, despite the fact that a large number of participants owned ALDs, only a small number have been trained in how to use them and thus only a few can utilize them in virtual communications. Therefore, self-advocacy in addition to the proper utilization of ALDs, and the implementation of communication strategies must be enhanced amongst CI recipients.

In addition, health care professionals and CI recipients should receive training on the different forms of emergency support that might be available, and how to provide/receive them. Again, the digitalization of health care services is expected to allow for a higher level of support during disaster scenarios, compared with traditional provision [30].

Finally, further research should be conducted to break down the classification of CI recipients even further. This will aid in understanding the diverse needs and consequently help to build a better, all-inclusive support system. Moreover, since the national CI societies and support groups were rated the highest in support provision during the pandemic, future investigative studies to examine benefits and initiate collaborative support approaches are recommended.

### Clinical implications

- Build a proper infrastructure for the successful integration of tele-rehabilitative approaches.
- Digitize re/habilitation programmes and create tele-applications in Arabic language to implement a patient-centred care model in Saudi CI programmes.
- Implement communication strategies and special programming protocols for hearing aids/implantable devices in order to enhance affected frequency ranges in virtual platforms.
- Invest in educational audiologists in all school systems in Saudi Arabia to ensure that the environment, and assistive technology equipment in virtual platforms are properly used for students with hearing impairment.
- Adapt a patient-centred care model in CI programmes by empowering CI recipients to manage and troubleshoot their devices.

## Limitations

This study provides a large sample size and a high number of examined variables. Yet, findings of this study could not be compared with other findings in Saudi Arabia due to the lack of existing national research in this area. It could be seen as a limitation that the adult sample is much smaller than the pediatric sample; however, this reflects the ratio of adult to pediatric CI recipients in Saudi Arabia.

## Conclusion

Collective findings of this study emphasize the benefits of integrating tele-practice and digital platforms in cochlear implant services. Further studies to assess models of digitalization in health care for CI recipients in their native language are still required prior to implementation. In addition, integrating a patient-centered care model in CI services will prove to be extremely beneficial for CI recipients. Such an approach will not only achieve better performance outcomes but help to reduce negative feelings regarding personal wellbeing. Similarly, incorporating empowerment and self-advocacy into the rehabilitation of CI recipients will reduce fear and anxiety by making recipients feel more in control as they become independent and less reliant on professional support in their daily lives.

## Data Availability

The data that support the findings of this study are available from the corresponding author, RA, upon reasonable request.

## Footnotes

1
*These are estimated numbers based on combined information from25 large national CI programs, CI local suppliers, and CI companies annual reports. National database is not available yet.*

2
*These are estimated numbers based on combined information from 25large national CI programs and CI local suppliers. National database is not available yet.*
